# The Role of Local, Distal, and Global Information in Latent Spatial Learning

**DOI:** 10.1037/xan0000017

**Published:** 2014-04

**Authors:** Kerry E. Gilroy, John M. Pearce

**Affiliations:** 1 School of Psychology, Cardiff University, Cardiff, Wales

**Keywords:** latent learning, spatial learning, cognitive map

## Abstract

In 4 experiments that investigated latent spatial learning, rats were repeatedly placed on a submerged platform in a corner of a square swimming pool with walls of different brightness. When they were subsequently released into the pool for a test trial in the absence of the platform, they spent the majority of time in the corner used for placement training—the correct corner. This effect was observed in Experiment 1, even when the test trial took place in a transformed version of the training arena. Experiments 2 and 3 indicated that the correct corner was identified by local cues based on the walls creating the corner. Experiment 4 demonstrated that distal cues created by the two walls that did not surround the platform during placement training could also be used to identify the correct corner. There was no evidence of learning about the relationship between global cues provided by the entire arena and the goal. The absence of the opportunity to develop instrumental, stimulus–response associations during placement training indicates that stimulus–stimulus associations acquired during this training were sufficient to guide rats to the platform when they were eventually released into the pool.

For more than 50 years it has been assumed that animals are able to identify the position of a goal with reference to landmarks that are some distance from it. Tolman (1948) attributed this ability for spatial learning to the development of cognitive maps, which not only contain information about the spatial relationship among salient landmarks, but also about the position of important goals relative to the landmarks. Similar ideas have been expressed by Doeller and Burgess (2008), Gallistel (1990), Morris (1981), and O’Keefe and Nadel (1978). A rather different proposal is that when they are at a goal, animals identify its location by taking a mental snapshot of at least some of the visible landmarks (Cartwright & Collett, 1983; Cheung, Stürzl, Zeil, & Cheng, 2008; Sheynikhovich, Chavarriaga, Strösslin, Arleo, & Gerstner, 2009). Subsequent journeys to the goal then depend on the animal moving in such a way that its current view becomes progressively similar to the snapshot. Such snapshots will necessarily include information about the spatial relationships among the various elements they contain but, in contrast to cognitive maps, the animal will be able to find the goal only if it can see at least a portion of the cues contained within the snapshot. Finally, it has been proposed that when a goal is encountered it is associated with the surrounding cues by means of stimulus–stimulus associations (Miller & Shettleworth, 2007; Pearce, 2009; Rhodes, Creighton, Killcross, Good, & Honey, 2009; White, 2008). These associations are assumed to develop in much the same way as the associations that are acquired during Pavlovian conditioning (e.g., Pearce, 1994; Rescorla & Wagner, 1972). It is further assumed that animals navigate toward their goal by heading for the combination of stimuli with the greatest overall associative strength. As opposed to the snapshot account, these theories do not assume that representations based on combinations of stimuli contain information about the spatial relationships among the stimuli (see George, Ward-Robinson, & Pearce, 2001).

Despite the differences among the above three accounts, it follows from all of them that animals are able to retain a record of a goal and the stimuli that surround it. For the sake of convenience we shall refer to this record as a spatial S–S* association, where the initial S refers to a representation of one or more landmarks visible from the goal, and S* refers to the goal itself. A problem with demonstrating the acquisition of spatial S–S* associations is that when an animal encounters a goal, it is likely to have made a response that led to its discovery. The reward of finding the goal might then strengthen the response and enable the animal to reach the goal on a subsequent trial by repeating the same response. In other words, animals may not acquire a spatial S–S* association. Rather, their success in reaching the goal may be a consequence of instrumental conditioning resulting in specific responses being made to specific cues (e.g., Hull, 1943). In view of this possibility, there is rather little evidence that shows unequivocally that animals do indeed acquire spatial S–S* associations (see Benhamou, 1996; Bennett, 1996; Mackintosh, 2002; Muir & Taube, 2004).

In an attempt to provide evidence that animals acquire these associations. Horne, Gilroy, Cuell, and Pearce (2012) examined whether latent spatial learning is possible (see also Gaskin & White, 2007). Rats were first allowed to view the layout of a rectangular swimming pool by repeatedly being placed on a platform in one of the corners. On the completion of this placement training, the rats were then allowed to swim in the pool for the first time, with the platform removed. They showed a clear preference for searching in the two correct corners—those with the same geometric properties as the corner where placement training took place—than the two remaining, incorrect corners. Horne et al. argued that because the rats had no experience of swimming in the rectangular pool prior to the test trial, their preference for the correct over the incorrect corners could not have been a consequence of the influence of previously acquired stimulus–response associations. Rather, they argued the placement training must have resulted in the development of S–S* associations that allowed the correct corner to be identified. Given this conclusion, an obvious question to ask is what type of information was encoded in the initial component of the association. The experiments described later were designed with this question in mind.

A possible answer to the foregoing question is that during their placement training rats identified the position of the platform with reference to what we shall refer to as *local* cues. In the case of the experiment by Horne et al. (2012), such cues would be created by the walls that form the corner in which the platform was located. A rat might learn, for example, that the platform is in a corner with a long wall to the left of a short wall, or that the platform is near the left-hand end of a long wall. Another possible answer is that rats identify the location of the platform with reference to *distal* cues, which would be created by the walls that do not belong to the corner housing the platform, and which would include all the corners except the one in which placement training was conducted. According to this analysis, animals might identify the corner with the platform as the one that is diagonally opposite a corner where a long wall is to the left of a short wall. A third possibility is that animals construct a *global* representation of the entire rectangular pool, and identify where the platform can be found with reference to its shape (e.g., Cheng, 1986; Gallistel, 1990). Such an account is in keeping with the view that animals are capable of forming map-like representations of the spaces they inhabit (e.g., Doeller & Burgess, 2008; O’Keefe & Nadel, 1978; Tolman, 1948). It is also in keeping with the proposal that rats navigate by means of mental snapshots that encompass the whole environment (e.g.Cheung et al., 2008), or a large proportion of it (Sheynikhovich et al., 2009).

In an attempt to understand the nature of the spatial representation revealed by their latent learning task, Horne et al. (2012) repeatedly placed rats on a platform in one corner of a rectangular pool before they were released into a kite-shaped pool for a test trial. The kite was constructed from the same walls as the rectangle, and the two corners created by a long and short wall were both set at right-angles (see Figure 1). Corner A in the rectangle represents the correct corner, and is equivalent to Corner A in the kite because they are both right-angles with a short wall to the left of a long wall. Thus the local cues created by Corner A in each environment are identical. If animals make use of local cues for identifying where the platform is situated in the rectangle then, on being placed in the kite, they should express a preference for Corner A over any other corner. In contrast, the rearrangement of the walls that do not create Corner A ensures that the distal cues visible from this corner in the kite are different to those viewed from Corner A in the rectangle. If animals relied on distal cues to identify where the platform was located in the rectangle, then they should not show a preference for Corner A in the kite. For similar reasons, if the position of the platform in the rectangle was identified solely by its shape, then the difference between the kite and the rectangle would make it difficult to identify Corner A as the correct corner in the kite. The test revealed a strong preference for Corner A in the kite, relative to any other corner, which implied that local cues were used to identify where the platform was situated in the rectangle. The results of the experiment do not allow us to conclude, however, that rats failed to learn about the significance of distal cues, or the overall shape of the arena, for finding the platform. They may have identified the position of the platform with reference to both types of cue, but the nature of the test trial prevented them from taking advantage of this information.[Fig-anchor fig1]

To explore further the role played by local, distal, and global cues in spatial S–S* associations, the present experiments adopted the placement training methodology of Horne et al. (2012), but this treatment took place in a square arena with distinctive walls, rather than in a rectangular arena. By using different combinations of distinctive walls, and by rearranging them for the test trials, we were able to draw more precise conclusions about the nature of the spatial information that is used to identify where a goal is located than has hitherto been possible.

## Experiment 1

**Figure 2 fig2:**
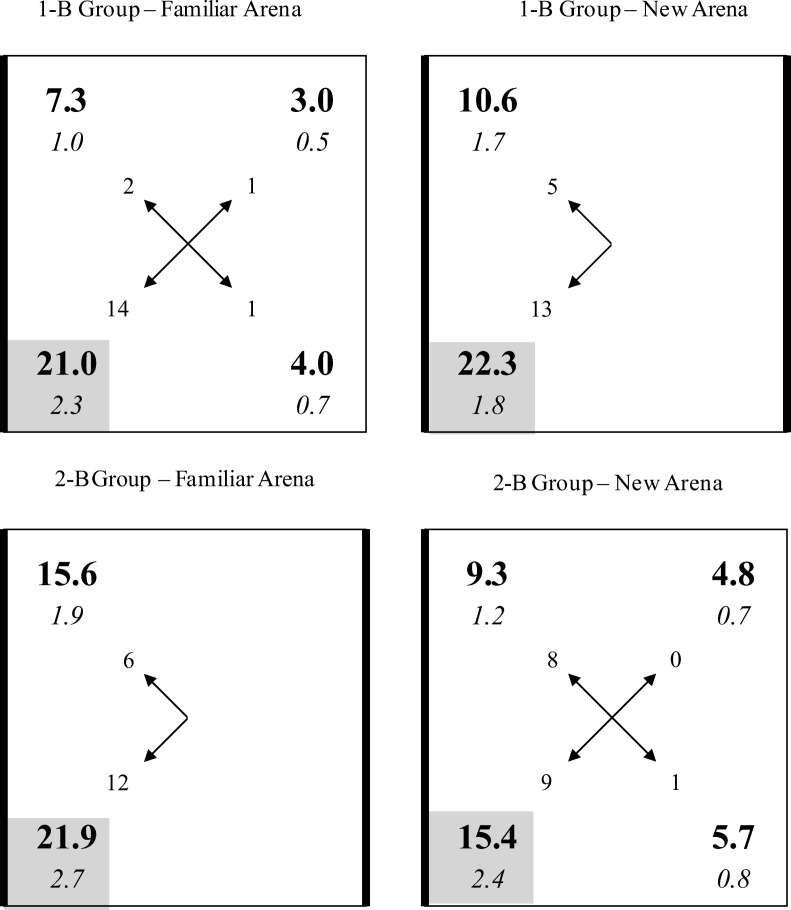
Group mean percentages (in bold) and standard errors (in italics) of the time spent in the corners of the familiar and new arenas during the test trials for the two groups of Experiment 4. The numbers at the end of the arrows indicate how many rats headed directly to each of the respective corners. The gray square indicates the correct corner, thick lines depict black walls, thin lines depict white walls.

The principal reason for conducting the first experiment was to confirm that latent spatial learning can be successful in a square arena in which some of the walls are different to the others. One group of rats was repeatedly placed on a platform that was situated in one corner of a square arena with three white walls and one black (B) wall—the 1-B group. The platform was always in the same corner at one end of the black wall (see upper left-hand panel of Figure 2). Toward the end of the placement training, two test trials were conducted in which the rats were allowed to swim in the pool for the first time, but with the platform removed. One test involved the familiar training arena, and one involved a new arena comprising alternating black and white walls (see upper right-hand panel of Figure 2). On the basis of the results of Horne et al. (2012) we expected rats to show a preference for the corner where the platform was previously situated over any other corner in the familiar arena. For the test in the new arena, it was not clear what the outcome would be. The training in the original arena might result in the position of the platform being identified by local cues based on the black and white walls creating the corner. For example, given the arrangement shown in the upper left-hand panel of Figure 2, rats might learn that the platform is situated at the left-hand end of the black wall, or in the corner where a white wall is to the left of a black wall (e.g., George et al., 2001). On this basis, a clear preference for the corners that match the one where placement training took place should be observed for the test in the new arena. As an alternative, rats might identify the position of the platform in the familiar arena with reference to distal or global cues. Because the changes required to create the new test environment will modify both types of cue, any control they acquire should be weakened, and result in animals displaying rather little interest in the corners that are common to those used for placement training.[Fig-anchor fig2]

A second group of rats was included in the experiment—the 2-B group. This group received the same placement training as the 1-B group, but in the arena with alternating black and white walls. The group also received the same two test trials as the 1-B group. Despite the use of a different training environment, the predictions made for the 2-B group are the same as for the 1-B group. Thus, if the corner where the platform was situated is identified by local cues, then the 2-B group will show a strong preference to search in the corners made from these cues in both arenas. If, however, the position of the platform is originally defined relative to distal or global cues, then replacing a black wall with a white wall in the new arena is likely to disrupt the influence of these cues and result in a rather weak preference for one type of corner over the other.

For all four experiments, any rat that fell from the platform during their placement training was discarded from the experiment, thereby ensuring that no rat had experience of swimming to the platform in the presence of the cues provided by the walls of the arena. We can thus be confident that the only knowledge that rats could use when searching for the platform during a test trial would be gleaned from their experience when viewing the arena from the platform. To encourage rats to remain on the platform during their exposure to the square arena, three preliminary sessions of placement training were given in a circular pool.

### Method

#### Subjects

The rats were 36 male, hooded-Lister rats purchased from Harlan Olac (Bicester, Oxford, England) weighing between 250 to 300 g at the start of the experiment. The rats were housed in pairs in a room with a 12-hr light–dark cycle, with the lights being turned on at 07.00. They had continuous access to food and water. The rats were assigned in equal numbers randomly to the two groups. All of the rats remained on the platform throughout the placement training.

#### Apparatus

A white, circular pool, 2 m in diameter and 0.6 m deep, was mounted on a platform 0.6 m off the floor in the middle of a room 4 m × 4 m × 2.3 m. The pool was filled daily with water made opaque by 0.5 L of white opacifer E308 (Roehm and Haas, Dewsbury, England) to a depth of 27 cm and maintained at 25 °C (± 2 °C). A white circular ceiling, with a diameter of 2 m, was suspended 1.75 m above the floor of the pool. In the middle of the ceiling was a 30-cm diameter hole, and 25 cm above the hole there was a camera with a wide-angled lens. Within the circular ceiling, eight, 45-W lights lit the pool from above. They were equally placed from each other in a 1.6 m diameter circle centered on the hole in the ceiling. The lights were 22.5 cm in diameter. The pool was surrounded by a white curtain that was 1.5 m high, and fell 25 cm below the edge of the pool. The curtain was attached to the circular ceiling. The room was also lit by two 1.53-m strip lights that ran end to end parallel to the floor at a height of 75 cm, on the east and west walls. In the center of a third wall there was a 1.75 m × 2 m door leading to an adjacent room. For the purposes of the experiment, the door was regarded as being south of the pool. The camera was connected to a computer and monitor in the room adjacent to the test room.

The two square arenas were constructed from four white Perspex walls measuring 141.0 cm long, 59.0 cm high, and 2 mm thick. Three of these walls also served as black walls by covering with matte black paint, on one side only, the region between the upper edge of the board and a horizontal line that was 24 cm above the surface of the water. The board was white below this line to reduce an unconditioned preference for black over white walls that we have found in preliminary studies. One side of these three walls could thus serve as a white wall whereas the other side could serve as a black wall. Each wall was placed vertically in the pool and suspended from square aluminum bars (2.0 cm × 2.0 cm) that extended beyond the edge of the pool. The walls were attached to the bars by nuts and bolts that enabled either side of each wall to face into the pool, with the bars outside the pool. Depending on the arrangement of the bars supporting the walls, the upper edge of the wall was either 48 or 50 cm above the surface of the water. The sequence in which individual boards were combined to create the arena varied randomly from session to session.

A circular platform, 10 cm in diameter, was used for the placement training. The surface was marked with concentric ridges to help rats grip the platform. The platform was mounted on a column that rested on the base of the pool. The surface of the platform was 2 cm below the surface of the pool. The platform was always on a notional line that bisected a corner of the pool, with the center of the platform at a distance of 25 cm from the corner.

#### Procedure

Rats were trained in groups of six. For 5 days a week, the rats were carried to the room adjacent to the test room in a light-tight box with six individual compartments. For each of the four trials of a session a rat was removed from the box and then, at the end of the trial, it was dried with a towel before being returned to the box. The remaining rats in the box were treated in the same way before the original rat was removed for its subsequent trial. The intertrial interval was approximately 5 min.

The pretraining took place in the circular pool in the absence of the black and white boards, and with curtain drawn around the pool. Rats were placed on the platform and expected to remain on it for 30 s. Any rat that left the platform was guided back by placing a finger in front of its snout and moving the finger slowly toward the platform. On returning to the platform, the rat was allowed to remain on it for the remainder of the 30 s. By the third session of pretraining, all the rats remained on the platform for the full 30 s. There were four possible positions for the platform, which were located on notional lines that bisected each of the four quadrants of the pool, and at a distance of either 25 cm or 50 cm from the edge of the pool. The position of the platform was randomized between trials with the stipulation that each quadrant was used once in each session and that the platform was 25 cm from the edge on two of the trials and 50 cm from the edge on the remaining two trials.

The 10 sessions of placement training took place in the square pool with three white walls and one black wall for the 1-B group, and alternating black and white walls for the 2-B group (see Figure 2). Each rat was repeatedly placed on the platform for 30 s facing the corner, in a corner created by a black and a white wall. For half the rats in each group the platform was always situated in a corner where a black wall was to the left of a white wall, and for the remaining rats the platform was situated in the corner where the black wall was to the right of the white wall. Within each session for the 2-B group, the platform was situated in one of the two possible corners for two trials, and in the other possible corner for the remaining two trials. The arena was rotated within the pool by 90, 180, or 270 degrees from one trial to the next in a random sequence, with the constraint that any given corner occupied four different locations, with reference to the experimental room, within each session.

The two test trials of the experiment were conducted on Trial 4 of Sessions 8 and 10 of the placement training. For half the rats, the first test trial took place in the arena that was used for placement training, and the second test trial took place in the new arena. The remaining rats received the opposite sequence of test trials. With reference to the experimental room, there were four possible orientations of the arena, and four possible positions for the experimenter to stand when releasing a rat. These factors were varied randomly, and independently of each other, for each rat in both test trials. The rats were released into the center of the pool, facing the experimenter. The experimenter always stood beside the center of a wall when releasing the rat, and then moved to the adjoining room to observe the rat on the monitor. The test trials were conducted in the absence of the platform, and rats were allowed to swim in the pool for 60 s.

#### Data analysis

The behavior of every rat was observed on the monitor connected to the camera throughout the experiment. During a test session, the rat’s movements were tracked on the computer, using Watermaze software (Morris & Spooner, 1990). For the purposes of analyzing the results from the test trials, circular search zones in each corner were used. The zones had a diameter of 30 cm with their centers located at a distance of 25 cm from the corner, equidistant from the walls creating the corner. The software recorded the percentage of the 60-s test trial that was spent in each zone. In addition, a record was taken of which corner the rat entered first after being released for the test trial. A corner was deemed to have been entered when the rat was less than 40 cm from the join between the walls creating the corner.

The analysis of results based on the time spent in different search zones was conducted with analyses of variance (ANOVA) using a rejection criterion of *p* < .05. The reported effect size for ANOVA with more than one factor is partial eta squared (η_p_^2^), whereas for comparisons between two means it is eta squared (η^2^). For both measures of effect size, 95% confidence intervals (CI) were computed using the method reported by Steiger (2004).

### Results and Discussion

For the first three experiments of this report, the corner used for placement training was constructed from a distinctive wall (either black or striped) and a white wall. Any corner that is identical to this corner will be referred to as a correct corner. Corners that are the mirror image of the correct corner will be referred to as an incorrect corner. Moreover, to simplify their presentation, the results from the test trials have been normalized by ignoring the counterbalancing of the locations of the correct and incorrect corners in each experiment.

During a typical placement trial, rats would spend the entire 30 s slowly turning around on the platform looking at different regions of the arena. Based on where their snout was pointing, the majority of the trial was spent looking at the walls creating the correct corner and, to a lesser extent, at the corner itself. When rats looked at the remaining two walls, for the majority of the time their gaze was directed toward the regions that were nearest to the walls creating the correct corner.

To investigate the influence of counterbalancing the location of the correct corner on the outcome of the experiment, a four-way ANOVA was conducted with the within-group factors of zone (correct or incorrect) and arena (familiar or new), and the between-groups factors of group (1-B or 2-B) and location (whether the platform was in a corner with a black wall to the left of a white wall, or in a corner with a white wall to the left of a black wall). The analysis revealed a significant Location × Zone interaction, *F*(1, 32) = 4.74, *MSE* = 120.60, η_p_^2^ = .13, 95% CI [.00, .34], but the main effect of location, and all the remaining interactions were not significant, *F*s(1, 32) < 1.35. Subsequent investigation of the significant interaction, using tests of simple main effects, revealed a significant effect of zone for both locations, *F*s(1, 32) < 4.54. In view of this pattern of results, the effect of location was ignored in the following analysis.

The group mean percentages of time spent in the corners of the arena for the test trials with the 1-B group are displayed in the upper row of Figure 2, and the results for the 2-B group are shown in the lower row. For both groups, the results in the left-hand column are from the test in the familiar arena, which was used for training, and the results in the right-hand column are from the test in the new arena. The numbers highlighted by a gray square depict the group mean percentage of time spent in the single correct corner during the tests in the arena with one black wall, and the combined time spent in both correct corners during the tests in the arena with two black walls. The figures in the incorrect black and white corner were calculated in a similar manner.

**Figure 3 fig3:**
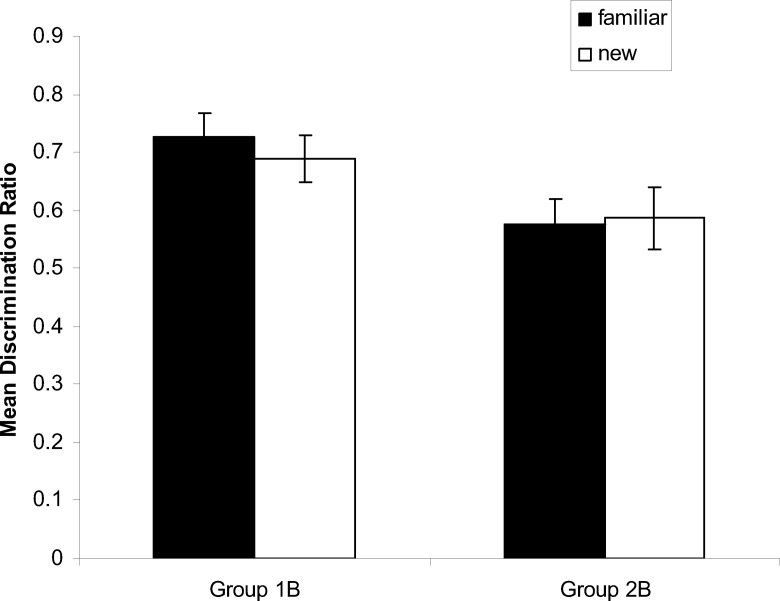
Group mean discrimination ratios and standard errors for the test trials with the two groups in the familiar and new test arenas of Experiment 1.

In each test for both groups, more time was spent in the correct corners than in any other corner, which confirms the effectiveness of the placement training. However, the discrimination between the correct and incorrect corners was more marked for both test trials with the 1-B group than with the 2-B group. This observation suggests that the critical influence on the outcome of the test trials was where training took place, and not where testing took place. To simplify the statistical analysis, a discrimination ratio was calculated for each of the two test trials for every rat. The ratio was of the form *C*/(*C* + *I*), where *C* is the percentage of time spent in the correct corners, and *I* the time spent in the other black and white corners—the incorrect corners. There were two correct and two incorrect corners for the 2-B group and one of each of type of corner for the 1-B group, which makes it meaningful to compare the two ratios directly. The results displayed in Figure 3 support the foregoing observation by showing that the discrimination ratios for the two test trials with the 1-B group are similar, and considerably greater than for the 2-B group that are also similar. A two-way ANOVA of individual discrimination ratios revealed a significant effect of group, *F*(1, 34) = 6.58, *MSE =* .043, η_p_^2^ = .16, 95% CI [.01, .37], but the effect of whether the test context was familiar or new, and the interaction were not significant, *F*s < 1.[Fig-anchor fig3]

Further support for the conclusion that placement training is more effective in an arena with one rather than two black walls comes from two sources. First of all, a series of ANOVA revealed that the 1-B group spent significantly more time in the correct than the incorrect corner in the arena with one black wall, *F*(1, 17) = 23.23, *MSE* = 73.04, η^2^ = .58, 95% CI [.21, .74], and in the arena with two black walls, *F*(1, 17) = 16.00, *MSE* = 76.67, η^2^ = .48, 95% CI [.30, .63]; whereas the 2-B group did not spend significantly more time in the correct than incorrect corners in the arena with two black walls, *F*(1, 17) = 2.24, *MSE* = 160.06, or one black wall, *F*(1, 17) = 3.66, *MSE* = 90.30. The second source derives from the analysis of the corners that rats headed to first on being released from the middle of the pool for a test trial. The number of rats that headed for a particular corner during each test trial are depicted by the numbers at the ends of the arrows in each panel of Figure 2. By way of example, 14 rats in the 1-B group headed directly for the correct corner during the test in the square with one black wall, whereas only two headed directly for the incorrect corner. Focusing on just those rats that headed directly for a corner at either end of a black wall, one-tailed binomial tests revealed that the number selecting the correct corner was significantly above chance for the 1-B group when tested in the arena with one black wall, *p* = .002, or two black walls, *p* = .048. Equivalent analyses for the 2-B group failed to reveal a significant effect for the test in the arena with one black wall, *p* = .50, or two black walls, *p* = .115.

To return to the analysis of discrimination ratios, the absence of a two-way interaction indicates that being tested in a new environment did not disrupt performance, relative to that seen by each group in the familiar environment. To assess further the effect of the transition from one environment to the other, we performed a Bayesian analysis, together with a standard paired *t* test, based on the discrimination ratios described earlier for every subject in the familiar arena and the new arena. The Bayesian analysis tells us whether the data favor more the null hypothesis (there being no difference between the discrimination ratios for the two environments) or the alternative hypothesis (there being a difference between the two sets of ratios). The Bayes factor is the relative probability of the null hypothesis to the alternative hypothesis such that a value of 1 would mean that each is just as likely. A value of 3 would mean that the null hypothesis is 3 times more likely than the alternative hypothesis given the data and the priors, and has been suggested as a cut off when deciding that data substantially favor the null hypothesis (see Rouder, Speckman, Sun, Morey, & Iverson, 2009, for more details). Analysis of the pairs of ratios found in favor of the null hypothesis, *t*(35) = 0.33, *p* > .05, Bayes factor = 7.41.

An implication of this conclusion is that the global properties of the training arenas were not important for identifying where the platform could be found. If these properties had been important, then changing them should have resulted in a reduction in the amount of time that was spent in a correct corner. The obvious caveat to this conclusion is that it is based on the failure to detect a drop in performance by each group when they were tested in the new rather than familiar arena (see Figure 3). Perhaps evidence of control by global cues would have been revealed with a different method of training or testing. That said, the experiment revealed that the placement training was highly effective when the 1-B group was tested in the new arena, which provided ample scope to observe a decrement in performance during this test trial.

For similar reasons, it is unlikely that the position of the platform was identified by means of distal cues in the 1-B group. These cues would be based on the two white walls that were opposite the correct corner, and would include the three corners that were not used for placement training. If they were used to identify where the platform was situated, then the replacement of one of these walls by a black wall to create the new test arena would be expected to remove some of the distal cues, and result in less time being spent in the correct corner during the test in the new than the familiar arena, which was not the case. Of course, it remains possible that for some reason rats relied on the distant white wall that was present throughout both tests as the distal cue for identifying where the platform was situated. Although there is a degree of special pleading with this account, we are unable to rule it out.

Finally, it seems most likely that rats identified the position of the platform with reference to local cues provided by the walls creating the corner where the platform was located for placement training. These cues were unaffected by the transformation of the familiar arena to the new arena for the 1-B group, which would then explain why performance was similar in both tests for this group. Many experiments have shown that rats can use a local cue to find a goal (e.g., Cheng, 1986; Pearce, Graham, Good, Jones, & McGregor, 2006), but it is possible this ability was based on previously acquired stimulus–response associations. The novel contribution of the present experiment is that it demonstrates the successful performance on the test trials was due to the influence of stimulus–stimulus associations involving the local cue.

The relative lack of success with the placement training for the 2-B group was not expected. The failure of this group to show a clear discrimination between the correct and incorrect corners in the familiar environment might be interpreted as evidence of a performance deficit, which was brought about by having to swim to a correct corner in the presence of two correct corners. The stumbling block for this suggestion is that the 1-B group showed a clear discrimination between the correct and incorrect corners in the arena with alternating black and white walls, which points to a failure of latent spatial learning in the 2-B group. Placement training, therefore, may be effective in some arenas, as used for the 1-B group, but not others, as used for the 2-B group. Before taking this conclusion seriously, however, it should be noted that both tests with the 2-B group revealed a numerical, but not statistically significant, preference for the correct over the incorrect corner. The possibility arises therefore that with a different and, perhaps, larger sample of rats, placement training would be effective in an arena with alternating black and white walls.

To test the foregoing possibility, a single group of rats in Experiment 2 received placement training and a test trial in the same environment as the 2-B group of Experiment 1. The size of the group was marginally larger than for its counterpart in Experiment 1. It was hoped that this factor, together with the change brought about by use of a different sample, would allow the experiment to reveal a clearer outcome to that seen in Experiment 1.

**Figure 4 fig4:**
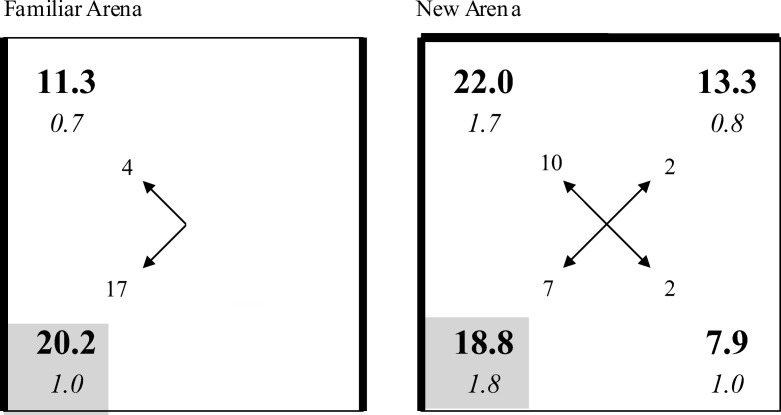
Group mean percentages (in bold) and standard errors (in italics) of the time spent in the corners of the familiar and new arenas during the test trials for Experiment 2. The numbers at the end of the arrows indicate how many rats headed directly to each of the respective corners. The gray square indicates the correct corner, thick lines depict black walls, thin lines depict white walls.

A second test trial was included in the experiment, which involved a square arena constructed from two adjacent black walls, and two adjacent white walls (see the right-hand panel of Figure 4). The purpose of this test relates to a further question posed by the earlier experiment. After placement training in an arena with one black wall and three white walls, the 1-B group showed a significant preference for the correct over the incorrect corners when it was tested in an arena with alternating black and white walls. We argued that this preference probably reflected the influence of spatial learning based on local cues, but the possibility that this preference was based on learning about the position of the platform with reference to a distal cue could not be ruled out. The test in the new arena in the present experiment involves a change to both of the distal walls that were present during placement training. If placement training is effective because it results in distal cues being used to indicate where the platform can be found, then the present test in the new arena will fail to reveal a preference for the correct over the incorrect corners. On the other hand, if the placement training encourages spatial learning based on local cues then because these cues will be present during the test in the new arena, a significant preference for the correct over the incorrect corners will be observed.[Fig-anchor fig4]

## Experiment 2

### Method

#### Subjects and apparatus

The 24 rats were from the same stock and housed in the same manner as for Experiment 1. Two of these were discarded from the experiment because they launched themselves into the pool from the platform during placement training, and one rat was discarded from the experiment because of a failure in the recording equipment during a test trial. The apparatus was the same as for Experiment 1.

#### Procedure

The details of the pretraining, the placement training, and the two test trials were the same as for the 2-B group of Experiment 1, except that the rats were given a total of 12 sessions of training, and the tests took place on the final trial of Session 10 and 12. The arrangement of the apparatus for the test in the new arena was different to Experiment 1 only in that it consisted of two adjacent white walls, and two adjacent black walls (see right-hand panel of Figure 4).

### Results and Discussion

To assess whether counterbalancing the location of the correct corner had an influence on the distribution of time spent in the correct and incorrect corners during the test trials, a three-way ANOVA was conducted with the within-group factor of zone (correct or incorrect), arena (new or familiar), and the between-groups factor of counterbalancing (whether the platform was in a corner with a white wall to the left or right of the black wall for placement training). The effect of counterbalancing, and all the interactions with this factor were not significant, *F*s(1, 18) < 3.40. This factor has therefore been ignored in the following analysis.

The left-hand panel of Figure 4 shows the group mean percentages of time spent in the correct and incorrect corners of the square pool during the test in the training arena. The results for the test in the new arena are shown in the right-hand panel. In both cases, the results have been normalized so that the correct corner, which is identified by the gray square, is created by a white wall to the left of a black wall. More time was spent in the correct black and white corner than the incorrect black and white corner in both arenas, with the extent of the preference being similar in both environments. In support of these observations a two-way ANOVA based on individual percentages of time spent in the correct and incorrect corners revealed a significant effect of corner (correct or incorrect), *F*(1, 20) = 34.92, *p* < .001, *MSE* = 31.0, η_p_^2^ = .51, 95% CI [.32, .77], but the effect of arena (familiar or new), *F* < 1, and the interaction, *F*(1, 20) = 1.35, *MSE* = 41.6, were not significant.

A striking result from the experiment is that during the test in the new arena more time was spent in the corner created from two black walls than any other corner, although this difference was small for the comparison with the correct corner. A one-way ANOVA based on individual percentages of time spent in each of the four corners of the new arena revealed a significant effect of corner, *F*(3, 60) = 15.1, *p* < .001, *MSE* = 53.1, η_p_^2^ = .43, 95% CI [.22, .55]. Paired comparisons then revealed that significantly more time was spent in the corner created from two black walls than either the corner created from two white walls, *F*(1, 20) = 45.8, *MSE* = 41.53, η^2^ = .70, 95% CI [.40, .81], or the incorrect corner, *F*(1, 20) = 18.1, *MSE* = 43.79, η^2^ = .47, 95% CI [.29, .62], but the comparison with the correct corner was not significant, *F* < 1.

The left-hand panel of Figure 4 shows that 17 out of 21 rats headed for a correct corner after being released for the test trial in the familiar pool, which was significant with a binomial test, *p* < .005 (one-tailed test). In the new arena, only seven rats headed directly for the correct corner, with 10 heading straight for the corner with two black walls.

The results from the test trial in the arena with alternating black and white walls revealed a clear preference for the correct over the incorrect corners. This effect was evident in the strong tendency for rats to head directly for a correct than an incorrect corner, on being released into the pool. It also was evident in the greater amount of time that was spent in the correct than the incorrect corners throughout the 60-s test trial. When they are taken together, the results from Experiments 1 and 2 indicate that it is not easy for rats to benefit from placement training in a square arena with alternating black and white walls, but it is not impossible. In support of this last conclusion, we can note that unpublished experiments from our laboratory also have revealed successful placement training in an environment with alternating black and white walls.

The placement training resulted in significantly more time being spent in the correct than the incorrect corner in the novel test arena with two adjacent black walls and two adjacent white walls. The transformation to the training arena for this test makes it unlikely that placement training was effective through the location of the platform being identified with reference to a global representation of the training arena. Likewise, because the transformation involved a change to the two distal walls, and not the two local walls, relative to where the platform was originally situated, it is unlikely that the observed preference was due to the platform’s location being identified with reference to distal cues. Instead, the results in the new arena provide strong evidence that placement training encourages spatial learning based on local cues.

A further finding from the test in the new arena was that rats spent a large proportion of the trial in the corner created by two black walls. One explanation for this outcome is that during their placement training, rats identified the position of the platform as being at a particular end of a black wall. For the arrangement shown in Figure 4, it would be the left-hand end of the black wall. On being tested in the new arena, this information would then lead rats to search either in the correct corner, or in the corner with two black walls. An alternative explanation is that rats on being released into an unfamiliar pool exhibit an unconditioned tendency to head for the darkest region. Experiment 3 was conducted to evaluate these rather different explanations.

## Experiment 3

**Figure 5 fig5:**
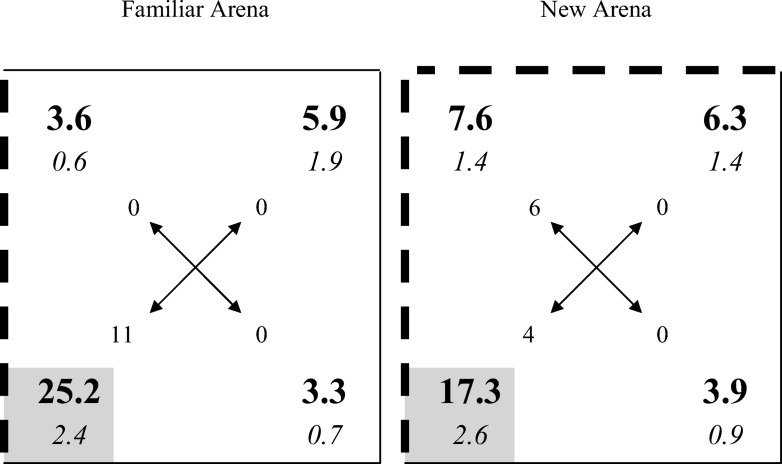
Group mean percentages (in bold) and standard errors (in italics) of the time spent in the corners of the familiar and new arenas during the test trials for Experiment 3. The numbers at the end of the arrows indicate how many rats headed directly to each of the respective corners. The gray square indicates the correct corner, dashed lines depict striped walls, thin lines depict white walls.

A single group of rats received placement training in the same manner as for the previous experiments, but in an arena with three white walls and one wall comprising vertical black and white stripes. The platform was situated in a corner created by a white wall and the striped wall. A test trial was then conducted in the familiar arena, to confirm the effectiveness of the placement training. A further test trial was conducted, which was based on the test in the new arena in Experiment 2, but with two striped walls rather than two black walls (see Figure 5). The vertical edges of the striped walls were both white, so that the corner created by the two striped walls was also white and thus lighter than the black corner in the new arena of Experiment 2. If the preference for the black corner in the previous experiment was due to an unconditioned preference for searching in dark corners, then conducting the equivalent test with striped walls should reduce this preference. As an alternative, if the preference for the black corner occurred because rats identified the position of the platform as being at a particular end of distinctive wall, it is possible that the present training will result in similar strategy being adopted. On this basis, the test in the new arena will reveal a strong preference for searching in the correct corner, and in the corner made from two striped walls.[Fig-anchor fig5]

Of course, the experiment might reveal a stronger preference for the correct corner over any other corner in the new arena. A straightforward explanation for this outcome is that during placement training rats will learn that the platform is in a corner where the striped wall is, say, to the right of a white wall. That is, latent spatial learning may result in an S–S* association in which the initial component involves structural information based on the spatial relationship between the two walls (e.g., Aggleton & Pearce, 2002; George et al., 2001).

### Method

#### Subjects and apparatus

The 12 rats were from the same stock and housed in the same manner as for Experiment 1. One rat was discarded from the experiment for failing to remain on the platform during one of the placement trials. The apparatus was the same as for the previous experiments, with the addition of two striped walls. The striped walls were made by attaching seven vertical strips of black plastic adhesive film to white walls. The stripes were 10 cm wide, separated by a gap of 10 cm, and extended from the top of the boards to below the surface of the water. There was a white stripe of between 5 and 6 cm in width at each edge of the striped walls.

#### Procedure

The details of the pretraining, placement training, and testing were the same as for the previous experiment except that the animals received a total of 11 sessions of training and the tests took place on the final trial of Sessions 8 and 11. During the placement training the pool was constructed from three white walls, and one striped wall. For half the rats the platform was in the corner with a white wall to the left of the striped wall, and for the remaining rats the platform was situated in the corner at the other end of the striped wall. The new arena for the second test trial was built from two adjacent white walls, opposite two adjacent striped walls.

### Results and Discussion

A similar analysis to that described for Experiment 2 was performed to examine the effect of counterbalancing where the platform was situated during placement training. The effect of counterbalancing, and all the interactions involving this factor were not significant, *F*s(1, 9) < 2.70, and this factor has therefore been ignored in the following analysis.

The mean percentages of time spent in each of the four corners during the test trial in the training arena can be seen in the left-hand panel of Figure 5, while the results for the test in the new arena can be seen in the right-hand panel. The results have again been normalized, so that for all rats the correct corner is depicted as the one in which a white wall is to the left of the striped wall. It is evident that the tests in both arenas resulted in a stronger preference for the correct corner than any other corner. To compare performance in the two arenas, a two-way ANOVA was conducted using individual percentages of time spent in the correct and incorrect corners. There was a significant effect of corner, *F*(1, 10) = 66.67, *MSE* = 44.02, η_p_^2^ = .87, 95% CI [.56, .92], but the effect of arena, *F*(1, 10) = 4.13, *MSE* = 18.5, and the interaction, *F*(1, 10) = 4.06, *MSE* = 76.0, were not significant. To compare the time spent in the four corners of each arena, separate one-way ANOVA were conducted. Analysis of individual percentages of time spent in each corner revealed a significant effect in the original arena, *F*(3, 30) = 38.20, *MSE* = 32, η^2^ = .79, 95% CI [.60, .85] and the new arena, *F*(3, 30) = 9.41, *MSE* = 40.53. η^2^ = .48, 95% CI [.17, .62]. Paired comparisons then revealed that significantly more time was spent in the correct corner than any other corner in the original arena, smallest *F*(1, 10) = 34.44, *MSE* = 59.47, η^2^ = .77, 95% CI [.34, .87], and the same was true for the new arena, smallest *F*(1, 10) = 8.05, *MSE* = 65.05, η^2^ = .45, 95% CI [.01, .68]. Further paired comparisons, based on the time spent in each of the three corners other than the correct corner, failed to reveal any significant differences for the original arena, *F*s(1, 10) < 1.34, *MSE* = 28.95, or the new arena, *F*s(1, 10) < 3.32, *MSE* = 21.75.

The placement training resulted in a clear preference for the correct corner over any other corner in both the familiar and the new arena. The results of the test trial in the new arena are of particular interest, as they have important implications concerning the nature of the local cues that were used to identify where the platform was situated. We noted in the introduction to the experiment that if the position of the platform is defined as being at a certain end of a striped wall, then rats will spend a similar amount of time in the correct corner and the corner with two striped walls. Moreover, more time will be spent in these corners than the remaining two corners. The pattern of results failed to confirm this prediction. Subjects, of course, might have identified the correct corner as being at a certain end of a white wall. If this were the case, then they should have spent a considerable amount of time in the corner created by two white walls in the new arena, as well as in the correct corner. Once again, the results failed to confirm this prediction. The possibility now remains that rats adopted both of the above strategies, which would result in them spending more time in corners built from either two striped walls, or two white walls, than the remaining incorrect corner. Once again, the results did not support this prediction. Therefore, if the position of the goal was identified with reference to local cues, then the most likely interpretation of our results is that the spatial relationship between the walls creating the correct corner was used to identify this corner.

The relatively small amount of time spent by subjects in the corner made from two striped walls stands in stark contrast to the amount of time spent in the equivalent corner for the test in the new arena in Experiment 2. Although this difference between the outcomes of the two experiments is consistent with the possibility that an unconditioned attraction to dark corners was responsible for the strong preference for the black corner in Experiment 2, it can be explained in other ways. The different rats that were used in the two experiments, or slight differences in the way they were trained, might have been responsible for the stronger preference that was seen to the corner created from two black rather than two striped walls.

The left-hand panel of Figure 5 shows that during the test in the familiar arena, all 11 rats headed directly to the correct corner on being released into the pool. A binomial test revealed that this preference for heading toward the correct than the incorrect corner was statistically significant, *p* < .001. The right-hand panel of the same figure shows that approximately half of the rats headed directly to the correct corner during the test in the new arena, and the remainder headed directly to the corner created by the two striped walls. Given that rats spent rather little time in this corner of the pool, it is unlikely that the tendency to head directly toward it was a consequence of what was learned during the placement training. Perhaps, therefore, rats were attracted to the corner created by two striped walls for no reason other than its novelty.

## Experiment 4

**Figure 6 fig6:**
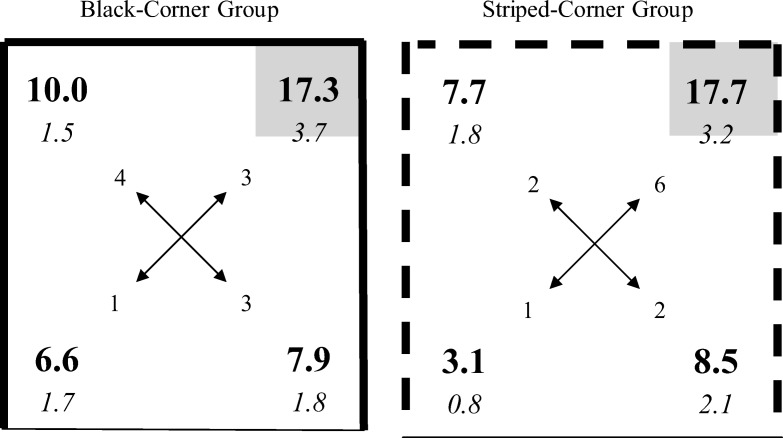
Group mean percentages (in bold) and standard errors (in italics) of the time spent in the corners of the familiar arena during the test trial for Experiment 4. The numbers at the end of the arrows indicate how many rats headed directly to each of the respective corners. The gray square indicates the correct corner, solid lines depict black walls, dashed lines depict striped walls, thin lines depict white walls.

The results thus far can be explained by assuming that placement training results only in local cues being used to identify where a platform is situated. The purpose of Experiment 4 was to determine if distal cues can also be used to reveal where a goal is hidden. The experiment involved two groups who were trained and tested in exactly the same way, but in different arenas to confirm the reliability and generality of any effect that was found. For the striped-corner group, three walls of the square arena were striped, and one was white. For the black-corner group three walls were black and one was white (see Figure 6). Placement training was conducted in a corner created by two walls of the same type—either two striped walls, or two black walls. As the arenas contained another corner that was identical to the correct corner, it would not be sufficient for subjects to rely just on local features to identify where the platform was situated. To identify the correct location of the platform, the animal would have to know about its relationship with the white wall on the far side of the arena from the corner where placement training was conducted. In other words, for placement training to be effective, it would be necessary to make use of distal cues. The experiment concluded with a single test trial in the same arena that was used for placement training.[Fig-anchor fig6]

### Method

#### Subjects and apparatus

The 24 rats were from the same stock and housed in the same manner as for Experiment 1. At the start of the experiment they were randomly assigned in equal numbers to the two groups. One rat from the black-corner group was discarded from the experiment for gaining experience of swimming in the pool during the placement training. One arena was constructed from one white wall and three black walls. In keeping with Experiment 1, the region of the wall that was painted black extended from the top of the board to 24 cm above the water. The other arena was constructed from one white wall and three striped walls, which were made in the way as for Experiment 3.

#### Procedure

The details of the pretraining and placement training were the same as for the previous experiments, with the following exceptions. The placement training took place in an arena with three striped walls and one white wall for the striped-corner group and in an arena with three black walls and one white wall for the black-corner group. Six rats from the striped-corner group received placement training in one of the corners created by two striped walls, and the remaining rats were trained in the other corner created by two striped walls. A similar method of counterbalancing was used for the black-corner group. There were eight sessions of placement training, with a single test trial occurring on Trial 4 of Session 8.

### Results and Discussion

To assess the influence of counterbalancing the location of the platform between the two corners made up from the same walls, a three-way ANOVA was conducted with the within-group factors of zone, the between-groups factors of arena (black and white walls or striped and white walls), and counterbalancing. The effect of counterbalancing, and all interactions with this factor were not significant, *F*s < 1. For the purposes of the following analysis, therefore, the factor of counterbalancing has been ignored.

In keeping with the previous experiments, the results from the counterbalanced conditions have been normalized to simplify their presentation. For the purposes of discussion, the corner that had the same appearance as the correct corner, but which never housed the platform during placement training, will be referred to as the incorrect corner. The right-hand panel of Figure 6 shows the results from the test trial with the striped-corner group, and the left-hand panel shows the equivalent results for the black-corner group. Both groups spent more time in the correct corner than any other corner during the test trial, and there was little difference between the performances of the two groups. In support of these observations, a two-way ANOVA, using individual percentages of time spent in each of the four corners, revealed a significant effect of corner, *F*(3, 63) = 9.17, *MSE* = 70.16, η_p_^2^ = .30, 95% CI [.10, .44] but the effect of group, *F*(1, 21) = 1.34, *MSE* = 14.04, and the interaction, *F*(3, 63) = 1.06, were not significant. Paired comparisons, using the results from both groups combined, revealed that significantly more time was spent in the correct corner than in the incorrect corner, *F*(1, 21) = 8.57, *MSE* = 97.15, η^2^ = .29, 95% CI [.16, .43], the diagonally opposite corner, *F*(1, 21) = 8.70, *MSE* = 110.52, η^2^ = .29, 95% CI [.16, .43], and the remaining corner, *F*(1, 21) = 18.03, *MSE* = 98.17, η^2^ = .46, 95% CI [.28, .61]. It is also evident from Figure 6 that the amount of time spent in the incorrect corner was similar to the amount spent in the remaining two corners. Further paired comparisons, using the results from both groups combined, revealed that the mean amount of time spent in the two corners created from two different walls did not differ significantly from the amount of time spent in the incorrect corner, *F*(1, 21) = 2.02, *MSE* = 28.91.

In contrast to the previous experiments, rats did not head in substantial numbers for a particular corner on being released into the pool. Analysis of the number of rats heading for each of the four corners, for the two groups combined, failed to reveal a significant preference for one corner over the others, χ^2^s(3) < 4.54, *p* > .20.

The stronger preference, in terms of the amount of time spent in the correct corner over any other corner by both groups confirms that rats relied on a distal cue to identify where the platform was situated. If they had relied solely on local cues then they would have been unable to tell the difference between the correct corner and the corner that, in terms of local features, was identical to the correct corner. Having established that animals can navigate with reference to distal cues, it becomes relevant to ask about the nature of these cues. Inspection of Figure 6 shows there were several cues that could have been used to identify where the platform was situated. Its location could have been defined, for example, as being diagonally opposite a corner where the white wall was in a particular spatial relation to the black (or striped) wall or, perhaps in a particular position with reference to the white wall. There is nothing in the present data that allows a choice to be made between these alternatives. It is also possible that rats acquired a global representation of the entire arena that was then used to find the platform on the test trial. We consider this possibility further in the General Discussion.

## General Discussion

All four experiments have shown that being placed on a platform in a corner of a pool with distinctive walls is a reliable method for demonstrating latent spatial learning. Such learning was effective when the distinctive walls were white and either black or striped. Such learning also was effective when the arena was constructed from one, two, or three identical walls. Initial attempts to demonstrate latent spatial learning in a swimming pool met with mixed success (e.g., Jacobs, Zaborowski, & Whishaw, 1989a, 1989b). In these earlier experiments the platform was located some distance from cues that could be used to indicate its location. It is possible that the successful demonstrations of latent spatial learning in the present studies, as well as those reported by Horne et al. (2012), were a consequence of the relevant cues being closer to the animals, and thus more salient, than in the earlier studies. Whatever the merits of this suggestion the previous results, along with those reported by Horne et al., demonstrate that latent spatial learning is a robust and reliable phenomenon.

Having established that latent spatial learning takes place, the main concern of the present article has been to identify the knowledge that results from being placed repeatedly on a platform in a corner of a pool with distinctive walls. In each experiment, steps were taken to ensure that rats did not gain any experience of swimming in the pool prior to their test trial. It is thus unlikely that performance during the test trials was a consequence of stimulus–response associations guiding the animals to the correct corner. Instead, it is more likely that the placement training resulted in the formation of spatial S–S* associations based on some or all of the stimuli visible from the goal (S), and the goal itself (S*). The experiments were intended to reveal the kind of information that is contained in the initial component of the association.

The results from the initial experiments, and in particular Experiment 2, indicate that local cues, by which we mean cues provided by the walls forming the corner housing the goal, play a prominent role in identifying where the platform is situated. Experiment 3 further suggests that the representation of these cues is based on structural information of the sort—white wall to the left of black wall (Aggleton & Pearce, 2002; George et al., 2001). It is possible that animals also learn that the platform can be found at a certain end of a particular wall, but the results from Experiment 3 revealed no support for this possibility.

Experiment 4 demonstrated that in addition to local cues, animals can use distal cues to identify the correct corner of an arena after placement training. By distal we mean the walls that do not create the correct corner, and the corners to which these walls contribute. An important issue that is raised by this finding concerns the manner in which animals treat local and distal information, when both can be used to indicate where a platform is located. It is tempting to suggest that animals will rely on local and distal cues at the same time, but one aspect of the results from Experiment 4 contradicts this suggestion. The test trials revealed a clear discrimination between the two corners that were identical in terms of their local cues, which must be attributed to rats making use of distal cues. If they also learned about the significance of local cues during placement training, then they should have expressed some interest in the incorrect corner during the test trial. There was no evidence of this being case. Perhaps, therefore, subjects learned about the significance of both local and distal cues during training, and chose to ignore the former during the test trial because they could be found in two different corners. As an alternative, the local cues provided by the incorrect corner may have been ignored during the test trial because they were in conflict with the information provided by the distal cues about where the platform was situated.

Another possible explanation for the results from Experiment 4 is that rats acquired a global representation of the entire arena that incorporated both local and distal information. There is something to be said in support of this possibility. The experiments have shown that rats made use of both local and distal cues, which would be expected if they have a global representation. The experiments also have shown that animals appreciate the spatial relationship between adjacent walls in the arena, which would be expected if they have a global representation of the arena. Furthermore, if rats acquired a global representation of the arenas in Experiment 4 then during the test trial they should be able to identify the correct corner, and treat the three remaining corners as being incorrect.

The results from Experiment 1, however, are not so compatible with the idea that animals acquire a global representation of their environment during placement training. Rats were trained in one environment, and then tested in a different environment that contained the same local cues that defined the correct corner in the original environment. Despite the overall differences between the two environments, there was no evidence that performance was disrupted by the transition from the familiar to the new arena. If knowledge about the location of the platform is based on a global representation, then changing the layout of the training arena should have affected performance on the test trial. The results of Experiment 1 thus make it hard to accept that that latent spatial learning depends on the development of global spatial representations.

One response to the failure of Experiment 1 to reveal any evidence of the influence of a global representation is to argue that the test was not sufficiently sensitive, or that the choice of arenas was not appropriate for some reason. Although this may be true, even if Experiment 1 had revealed a disruptive effect of testing rats in a new arena, such an outcome would not confirm that animals acquire global spatial representations. Instead, during training animals may identify the platform’s location with reference to one or more individual distal cues. The transformation of the arena for the test trial would then affect the ability to identify the correct corner by removing at least some of these cues. In other words, if any experiment should reveal that a change to an environment disrupts the search for the correct corner, then this finding can be attributed to the removal of a distal cue that was used to identify the correct corner. It is hard to know what evidence could be sought to go one step further and argue that the distal cue belonged to a global representation.

The results from our experiments are consistent with the suggestion that when they are at a goal animals acquire a mental snapshot (e.g., Cartwright & Collett, 1983; Cheung et al., 2008) or template (e.g., Haselgrove, George, & Pearce, 2005) of the surrounding landmarks. One advantage of this strategy is that it provides a simple means for representing the spatial relationships between the landmarks incorporated into the snapshot. If the goal is located in a black and white corner of a square with one black wall and three white walls, then a snapshot of the correct corner would provide all the necessary information to discriminate between the correct corner and its mirror image. According to certain authors (e.g., Aggleton & Pearce, 2002), mental snapshots are likely to be based on a restricted view of the environment, such as that provided by local cues. If this account is correct then it is quite easy to understand how it could be used to guide animals toward the correct corner when released into the pool for the first time. On being released, rats might have referred to the mental snapshot taken during placement training, and then swim in a manner that ensured the current view of the pool became progressively more similar to the remembered image (e.g., Cartwright & Collett, 1983; Sheynikhovich et al., 2009; Stürzl, Cheung, Cheng, & Zeil, 2008). With this account in mind, it is noteworthy that Experiments 1 and 2 revealed that, on being released for a test trial, the majority of rats headed directly for the correct corner in either the familiar or the new arena. We argued that in both of these experiment rats relied on local cues and, when used in the manner just described, a mental snapshot based on these cues would guide rats directly to the correct corner.

Turning now to Experiment 4, we argued that the correct corner was identified by reference to distal, rather than local cues. In theory it should be possible to navigate by means of distal cues in the manner just described, but it appears that this strategy was not adopted because rats did not swim in substantial numbers directly to the correct corner on being released into the pool. When released from the middle of the pool, it may be rather difficult to use a mental snapshot based on distal cues because it involves moving away from the remembered cue, rather than toward it. At the outset of the test trial this difficulty may then have left subjects with no alternative but to select corners at random until they found themselves in a corner where the view of the distal cues matched their mental snapshot. At this point they may then have been encouraged to remain in the corner and search for the platform.

Cheung et al. (2008) proposed that mental snapshots are global in nature and involve a panoramic view of the entire apparatus (see also Sheynikhovich et al., 2009; Wystrach, Cheng, Sosa, & Beugnon, 2011). It is not easy to derive precise predictions from these formal accounts concerning the present experiments, but it is possible that the findings from Experiment 4 will be hard to explain with them. For both groups, it seems likely that the panoramic view from the correct corner will be quite similar to the view from the incorrect corner containing the same local cues as the correct corner. The view from the foregoing incorrect corner is also likely to be rather different to the panoramic views from the remaining two corners. On this basis, therefore, it might be thought subjects would spend more time during the test in the incorrect corner made from two identical walls than in the remaining two incorrect corners. We already noted that the results from the experiment lend no support to this prediction. Furthermore, if animals navigate by means of global snapshots then the changes made to the training arenas for the test trials of Experiment 1 would be expected to weaken the ability to find the correct corner. As noted already, evidence to support this prediction was not forthcoming. Both of these arguments, however, are based on a null result and should be treated with a measure of caution.

A more general problem with the proposal that animals navigate by means of snapshots concerns findings from experiments that have tested for cue competition effects in spatial learning. If an animal is required to find a goal in the corner of a distinctively shaped arena, such as a rectangle or a kite, then given the appropriate training, the presence of features attached to the walls can overshadow, block, potentiate (e.g., Pearce et al., 2006), or even supercondition (Horne & Pearce, 2010) the control acquired by the cues provided by the shape of the environment. Overshadowing can readily be understood with a snapshot account of animal navigation. The removal of the overshadowing cue for the test trial will mean that the snapshots acquired during training will no longer match very well any of the views of the apparatus during testing, and make it difficult for the correct location to be identified (Wystrach & Graham, 2012). The remaining phenomena, however, are not so readily explained. In particular, it is hard to understand why the removal of a feature in a demonstration of potentiation or superconditioning should enhance the control acquired by cues provided by the shape of the environment.

Phenomena such as potentiation, blocking, and superconditioning can be explained by an associative analysis of spatial learning (e.g., Pearce, 2009), which suggests that such an analysis might also provide a satisfactory account for the outcome of the present experiments. That is, by placing a rat on a platform, the nearby distinctive cues might acquire associative strength that will attract the rats to the cues during the test trial. There are, however, at least two problems with this account. One such problem is posed by the results of Experiment 3, which revealed that rats identified the correct corner by referring to its structural properties or, in other words, the spatial relationship between the walls creating the correct corner. As noted in the Introduction, current theories of learning do not provide a mechanism that allows this type of relational information to be encoded associatively (but see George et al., 2001). At the very least, therefore, the present results indicate that an associative analysis of spatial learning will need to provide an account of how animals encode structural information if it is to explain fully our results. A further problem for an associative analysis of spatial learning is posed by the results of Experiment 4. If associative learning is based solely on local cues then it would not have been possible for rats to show a preference for the correct corner over the incorrect corner, both of which were identical in terms of their local features. One plausible solution to this problem is to suggest that the rats identified the position of the platform with reference to a configuration (e.g., Pearce, 1994) that encompassed both local and distal cues. By searching in the pool at random, until they came across this configuration, rats during the test trial would eventually find themselves in the correct corner. Of course, the configuration would need to take account of the manner in which the pool was structured for this strategy to be effective.

It would appear, therefore, that the representation of S, in an S–S* association is based on a template (Haselgrove et al., 2005) or snapshot Cartwright and Collett (1983) of at least a part of the environment. The rules governing the strength of this association could then be similar to those found in a configural theory of learning. The theory of Pearce (1994), for example, not only provides an account of how learning based on configurations develops, but also provides an account of how effects such as blocking and superconditioning can be found with components of configurations.

## Figures and Tables

**Figure 1 fig1:**
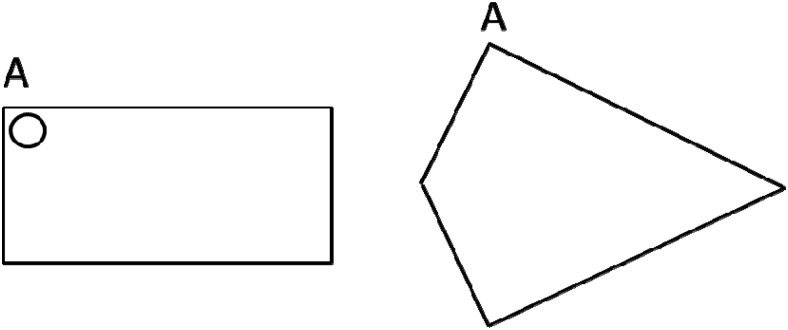
Plan of the apparatus used by Horne, Gilroy, Cuell, and Pearce (2012) to investigate the transfer of latent spatial learning from a rectangle to a kite-shaped arena. The circle depicts the platform.
